# Durability Assessment of Bonded Piezoelectric Wafer Active Sensors for Aircraft Health Monitoring Applications

**DOI:** 10.3390/s24020450

**Published:** 2024-01-11

**Authors:** Jesús N. Eiras, Ludovic Gavérina, Jean-Michel Roche

**Affiliations:** DMAS, ONERA, Université Paris-Saclay, F-92322 Châtillon, France; ludovic.gaverina@onera.fr (L.G.); jean-michel.roche@onera.fr (J.-M.R.)

**Keywords:** piezoelectric wafer active sensor, guided waves, structural health monitoring

## Abstract

This study conducted experimental and numerical investigations on piezoelectric wafer active sensors (PWASs) bonded to an aluminum plate to assess the impact of bonding degradation on Lamb wave generation. Three surface-bonded PWASs were examined, including one intentionally bonded with a reduced adhesive to create a defective bond. Thermal cyclic aging was applied, monitoring through laser Doppler vibrometry (LDV) and static capacitance measurements. The PWAS with the initially defective bond exhibited the poorest performance over aging cycles, emphasizing the significance of the initial bond condition. As debonding progressed, modifications in electromechanical behavior were observed, leading to a reduction in wave amplitude and distortion of the generated wave field, challenging the validity of existing analytical modeling of wave-tuning curves for perfectly bonded PWASs. Both numerical simulations and experimental observations substantiated this finding. In conclusion, this study highlights the imperative of a high-integrity bond for the proper functioning of a guided wave-based structural health monitoring (SHM) system, emphasizing ongoing challenges in assessing SHM performance.

## 1. Introduction

Nowadays, aircraft health assessments heavily rely on periodic nondestructive testing inspections, scheduled at fixed intervals as part of preventive maintenance operations. This practice is labor-intensive, expensive, and requires immobilizing the aircraft during the inspection. Structural health monitoring (SHM) technologies offer a promising alternative, allowing for improved maintenance practices that increase aircraft availability and prevent unscheduled maintenance events [1,2]. Currently, such events contribute significantly to flight delays and impose substantial economic burdens. Despite the acknowledged benefits that SHM may bring to the aeronautical industry, the adoption of these technologies remains limited [3,4]. One major concern is related to the durability of SHM systems in the harsh environmental conditions encountered by airborne hardware, including extremely high or low temperatures, dynamic and transient loads, humidity, and radiation. These conditions can impair the efficacy of SHM systems, ultimately leading to a loss of effectiveness in informing on the structural condition. Therefore, a thorough reliability analysis of SHM systems is of foremost importance to justify the practical implementation of SHM in the aeronautical industry [5].

Piezoelectric wafer active sensors (PWASs) stand out as a pervasive sensing technology in SHM applications, distinguished by their lightweight construction, low-power energy consumption, and ability to be mounted on structures without compromising their mechanical integrity. Techniques such as electromechanical impedance and ultrasonic-guided wave inspections rely on PWASs and find widespread use in SHM for aerospace structures in the literature [6,7,8]. In general, the long-term durability performance of such SHM techniques hinges on the creation of a high-integrity bond between the PWAS and the structure under inspection. A lack of understanding about the bonding integrity and its evolution with time may lead to false calls and inaccurate assessments. Concerns regarding the bonding integrity with operating time are already highlighted in the SAE 6461 guidelines [9], emphasizing the crucial need to maintain a high-integrity bond between sensors and the monitored structure throughout its lifetime. In terms of sensor durability, the SAE 6461 relies on the environmental testing procedure required for the qualification of airborne hardware specified in the RTCA DO-160 [10]. Still, the SAE 6461 recommendations lack specificity when it comes to detailing the test practices and acceptance criteria for evaluating sensor performance.

Concerning bonded PWASs, thermal coefficient mismatch, mechanical strain compatibility [11,12,13], and the thermal stability of the adhesive [14] stand out as significant degradation mechanisms. In addition, PWASs commonly face issues such as sensor cracking, soldering breakage, and a decline in piezoelectric properties with aging [15]. These concerns have been highlighted through various tests aimed at promoting degradation mechanisms on mounted and free PWASs, including impact loading [16,17], quasi-static and fatigue loading [18,19,20], vibration [19,21], pressurization (altitude) [19,20], radiation [22,23,24], cryogenic [18,25,26], and high-temperature cycling [18,19,21,27], as well as humidity and submersion in diverse fluids [18,20]. In all these instances, electromechanical impedance (EMI) measurements were the preferred testing method for assessing the performance of PWASs upon accrued damage. Specifically, as the debonding area between the PWAS and the host structure increased, the real and imaginary parts of the impedance spectra exhibit differential evolutions owing to modifications in the boundary conditions enacted by the adhesive layer. Moreover, the physical degradation of the PWAS was likewise discernible in the EMI spectra. In practical terms, various error metrics (e.g., root-mean-square deviation) related to the initial (pristine) EMI measurement have been employed to identify faulty sensors [13,28,29]. However, such metrics may not consistently exhibit a monotonically increasing trend with sensor degradation. The most prevalent approach involves examining the evolution of impedance near the quasi-static regime [17]. As explained in [17], this measurement independently conveys information about the sensor’s health, virtually irrespective of the structural condition, allowing for discrimination between PWAS debonding or physical degradation of the PWAS (e.g., cracking and loss of piezoelectric properties). Notably, the main advantage of EMI is that it can be seamlessly integrated into in-service structures, enabling a self-diagnostic capability for PWASs. However, for practical SHM implementation in aircraft, careful consideration and compensation for the effects of the operating environment are essential. Otherwise, inaccurate assessments can be obtained. Commonly, the operating temperature, vibrations, and bearing load have a meaningful effect on the electromechanical behavior of bonded PWASs [30,31,32,33,34].

Moreover, in applications where PWASs are intended for Lamb wave-based inspection, their performance evaluations often involve pitch–catch measurements. Experimental evidence obtained elsewhere has reported variations in the amplitude and phase of the received Lamb waves upon the degradation of the PWAS bonding, yet showing no consistent trends with excited frequency and extent of degradation [27,35,36,37,38]. For uniformly bonded PWASs, the elastic properties and thickness of the adhesive layer significantly influence emitting and receiving performance, as indicated by existing analytical modeling based on the shear lag theory [39,40,41]. At higher frequencies, the eventual interaction with PWAS resonances produces variations in the amplitude of Lamb-wave modes with bonding layer properties that are not well captured through such analytical modeling [42,43]. This phenomenon becomes more intricate when PWAS debonding occurs, modifying the boundary conditions of the PWAS and exacerbating the interaction with PWAS resonances.

To evaluate sensor health, another common practice in ultrasonic pitch–catch measurements involves conducting reciprocity checks while analyzing the behavior of a sensor network consisting of identical PWASs. In the latter case, any break of the reciprocity principle may be informative of sensor health [44]. In other instances, specifically in laboratory condition measurements, laser Doppler vibrometry (LDV) scans were used to assess the generated wave field [11,21,27,28,45]. This method allows for comprehensive imaging of the generated wave field, enabling the discernment of waves trapped within the PWAS due to local debonding. In exceptional cases, remarkable outcomes have been achieved through the application of advanced ultrasonic testing techniques, including scanning acoustical microscopy [18,22] and near-field ultrasonic scattering [11], to finely diagnose PWAS cracking and debonding in laboratory settings.

In this study, we investigated the effects of piezoelectric wafer active sensor (PWAS) bonding degradation on Lamb waves and electromechanical impedance using a combination of experiments and numerical simulations. The experiments involved electrical impedance and laser Doppler vibrometry (LDV) measurements, performed on a PWAS mounted on an aluminum plate that underwent thermal cycling between −55 °C and 85 °C. It was expected that the thermal aging effect produces a progressive debonding of the PWAS. A finite element model (FEM) was configured to reproduce the effects of debonding on the resulting electromechanical behavior of the PWAS. Indeed, existing mathematical modeling for wave generation (tuning curves) [41,46,47] or electrical impedance of PWASs [48,49] do not account for non-uniform distributions of the PWAS/structure bonding interface, thus transforming the analysis into a primarily numerical problem. Finally, conclusions were drawn on the potential effects that a degraded bonding may involve on the performance of a guided wave SHM system.

## 2. Materials and Methods

### 2.1. Materials and Accelerated Aging

**Figure 1:**
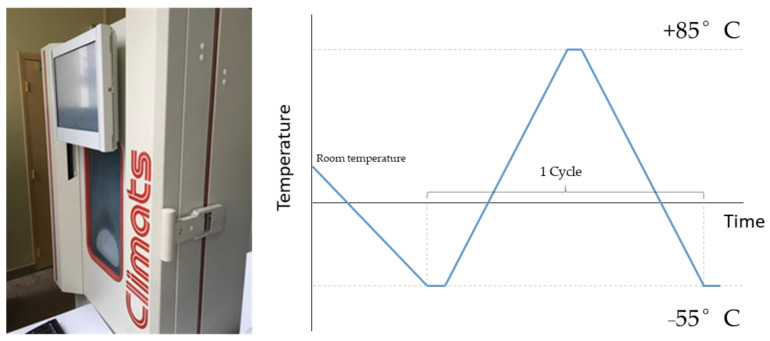
Schematic representation of the accelerated testing procedure in a climatic chamber.

Three piezoelectric lead–zirconate–titanate (PZT) sensors (Fuji C6, dimensions: diameter—20 mm and thickness—200 μm) were glued with a cyanoacrylate-based glue (Loctite^®^ 454 (Düsseldorf, Germany)) onto a squared aluminum plate measuring 500 mm × 500 mm × 2.4 mm. Before applying the glue, the aluminum substrate was thoroughly cleaned with acetone and prepared with a surface neutralizer. Two PWASs (denoted henceforth as D1 and D2) were affixed to the aluminum plate with an adequate amount of cyanoacrylate glue to produce a uniform bond. The third one (denoted as D3) was purposely affixed with an insufficient amount of an adhesive to produce a defective (non-uniform) bond. The aluminum plate was then subjected to accelerated aging cycling according to the temperature extremes instructed by the standard DO-160 [10]. The aging cycle consisted of an initial preconditioning step from ambient temperature (~23 °C) to −55 °C in a climatic chamber and subsequent repeated cycling between −55 °C and 85 °C at a constant rate of ±3 °C/min. The temperature was held for 10 min in between the temperature extremes; hence, a complete cycle lasted 1 h and 53 min. Figure 1 shows a schematic of the thermal aging protocol. The performance of the PWAS was initially evaluated at room temperature and after ~500 h (350 cycles) and ~750 h (525 cycles) of exposure to the accelerated aging conditions. The aluminum plate was conditioned at room temperature before testing.

### 2.2. Inspection Techniques

#### 2.2.1. PWAS Self-Diagnostics

Electrical impedance measurements were used to monitor the integrity of the PWAS bond through the thermal aging treatment. The admittance (inverse impedance) of a bonded PWAS (*Y_bonded_*) can be expressed using the following convolution:

(1) $$Y_{bonded}\left(\omega \right)=Y_{free}\left(\omega \right)\cdot H\left(\omega \right)$$

where *Y_free_* is the admittance of the free PWAS, and the function *H*(ω) depends on the piezoelectric, mechanical, and dimensional properties of the mechanical system (PWAS, substrate, and adhesive). Different analytical expressions for *H*(*ω*) can be derived from the analytical modeling of bonded PWASs provided elsewhere [31,48,49]. It follows from these studies that any change affecting the integrity of the system during thermal aging (PWAS, adhesive, and substrate) is manifested in the admittance spectrum. At the low-frequency range (usually < 1 kHz), the PWAS is treated as a pure capacitor; hence, $Y_{free}=i\omega C_{free}$ and $Y_{bonded}=i\omega C_{bonded}$. In most cases, from comparison to static capacitance measurements of healthy bonded PWASs, one can detect PWAS debonding as the capacitance increases (in general, *C_free_* > *C_bonded_*), or a decrease in PWAS performance (e.g., breakage or decline in piezoelectric property) as the measured capacitance decreases below *C_bonded_*, hence providing a basis for diagnosing PWAS health. Herein, the static capacitance values of the bonded PWAS were evaluated at 100 Hz using an HP4194A impedance analyzer apparatus. The values of the free PWAS were also measured for reference.

#### 2.2.2. Laser Vibrometry

**Figure 2:**
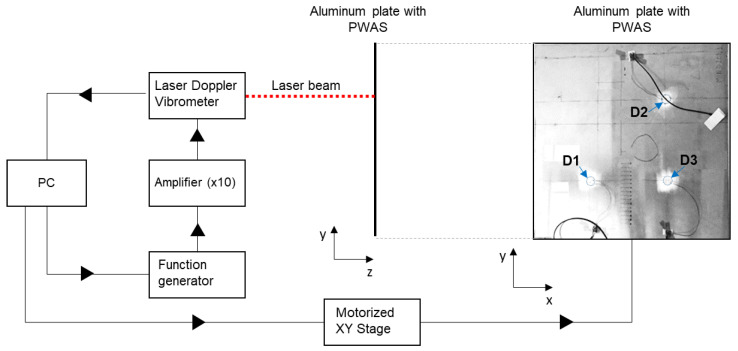
Wave field imaging testing configuration using a laser Doppler vibrometer. The positions of the three PWASs (D1, D2, and D3) are indicated with arrows.

A 25-kHz tone burst of 5 cycles was generated with an arbitrary function generator (Keysight 33500B (Santa Rosa, CA, USA)) and fed to an ×10 amplifier, resulting in an output voltage amplitude of 40 Vpp. The plate was mounted on a testing rig comprising two micro-controlled linear positioning stages that allowed for the horizontal and vertical displacement of the laser spot over the inspected zone. The out-of-plane vibration promoted by every PWAS into the aluminum plate was sensed with a Polytech OFV-505 laser Doppler vibrometer. One scan of 10 mm × 10 mm with a spatial resolution of 0.5 mm^2^/pixel was effectuated over a zone covering every PWAS. Figure 2 shows a schematic representation of the testing setup.

In addition, the wave field scans obtained experimentally were post-processed to obtain a proxy of the debonded area across different aging stages. The post-processing procedure involved extracting the root-mean-squared (RMS) amplitude at each pixel, applying min–max normalization to the resulting scan, and binarizing it according to an arbitrary threshold. Notably, the outcome of this analysis depends on the selected threshold, meaning that different results would be obtained by choosing different threshold values. Appropriate threshold selection must be based on novelty detection using pristine RMS scans as a reference (those initially obtained for D1 and D2). Specifically, herein, normalized RMS amplitudes higher than 15% were assigned a value of 0 (debonded), while all other values were set to 1 (bonded). A set of dilate/erode operations was applied to the resulting binary image to achieve a more accurate representation of the debonded zone. The threshold value was determined empirically after the careful examination of the LDV scan histograms. Through this filtering process, the extent of PWAS debonding was estimated. The outcome of this analysis was subsequently used to configure the spring elements that were used to represent, in the FEM, the adhesive layer in between the PWAS and the aluminum plate so that only the identified remaining bonded area retains the initial (pristine) adhesive mass and stiffness.

### 2.3. Finite Element Modeling

**Figure 3:**
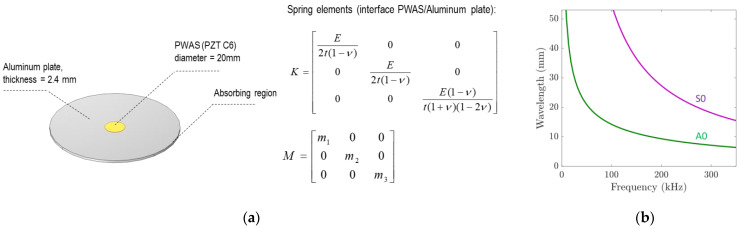
(**a**) Schematic representation of the FEM and (**b**) Lamb wave dispersion curves for the considered aluminum plate.

The effects of a degraded adhesive bond contact between one PWAS and the aluminum plate were further analyzed using the finite element method (FEM) in COMSOL Multiphysics. Figure 3a presents the geometry and boundary conditions of the 3D FEM model. This model consists of an infinite elastic plate (region of study of 100 mm × 100 mm × 2.4 mm) and a PWAS (radius: 10 mm; thickness: 0.2 mm) mounted on the top of the plate surface. For a Fuji C6 ceramic, the strain–charge piezoelectric matrix (D) and relative permittivity matrix (e) were set to [50]:

(2) $$D=\left[\begin{array}{ccc} 0 & 0 & 0\\ 0 & 0 & 0\\ -210 & -210 & 472 \end{array}\;\;\begin{array}{cc} 0 & 758\\ 758 & 0\\ 0 & 0 \end{array}\right]\mathrm{pC}/N$$

and

(3) $$e=\left[\begin{array}{ccc} 2270 & 0 & 0\\ 0 & 2270 & 0\\ 0 & 0 & 2130 \end{array}\;\;\right]$$

The density of the PWAS was set to 7650 kg/m^3^. The use of interfacial spring elements allows for the phenomenological modeling of bonding deterioration in the FEM [51]. Interfacial spring elements with a diagonal mass matrix M were used to model the attachment between the PWAS and the aluminum plate. The normal and tangential stiffness of the interface elements were obtained from the thickness (*t*), elastic properties (Young’s modulus (*E*)), and Poisson’s ratio (*ν*) of the interface layer as:

(4) $$K=\left[\begin{array}{ccc} \frac{E}{2t\left(1-\nu \right)} & 0 & 0\\ 0 & \frac{E}{2t\left(1-\nu \right)} & 0\\ 0 & 0 & \frac{E\left(1-\nu \right)}{t\left(1+\nu \right)\left(1-2\nu \right)} \end{array}\right]$$

The typical properties of cyanoacrylate-like adhesives were considered. The Young’s modulus, Poisson’s ratio, and density of the adhesive were set to 1 GPa, 0.30, and 1100 kg/m^3^, respectively. The thickness of the adhesive layer was set to 100 µm. These properties were considered constant for pristine and aged scenarios while varying the extent of the bonded area beneath the PWAS according to the identified areas from the post-processing conducted on the LDV scans (as described in Section 2.2.2). According to this post-processing stage, only the identified remaining bonded area retained the initial (pristine) adhesive mass and stiffness (and set to zero elsewhere).

An electrical potential of 1 V was applied on the topmost surface of the PWAS, and its bottom surface was grounded. Then, the harmonic response was evaluated from 10 kHz to 350 kHz at 1-kHz steps. A reflectionless absorbing region flanking a region of study centered on the PWAS position was configured to simulate an infinite plate [52,53,54]. According to the method described by Ke et al. [54], the volumetric mass (*ρ^AR^*) and elastic properties (*C_ij_^AR^*) of the artificial domain were set to evolve with the distance (*r*) to the region of study as:

(5) $$C_{ij}^{AR}=C_{ij}^{plate}\cdot \alpha$$

(6) $$\rho^{AR}=\rho^{plate}/\alpha$$

where the function *α*(*r*, *L*) weighs the mass and the elastic properties of the plate as:

(7) $$\alpha =1-D\left(r\right)+iD\left(r\right)$$

and wherein the function *D*(*r*) reads as [54]:

(8) $$D\left(r\right)=A{\left(\frac{r}{L}\right)}^{3}$$

wherein *A* is a scaling parameter herein set to 1.5, and *L* is the size of the absorbing region. *L* was set to 1.5 times the maximum wavelength propagating in the plate (*λ_max_*), which is an adequate size to fully obliterate the propagating waves through the absorbing region [54]. Obtaining the plate dispersion curves is therefore instrumental to configuring the absorbing region. These were obtained through solving Lamb’s equations with the help of the software Dispersion Calculator v2.2 [55]. Figure 3b shows the obtained Lamb wave dispersion curves, considering the typical elastic properties and density of an aluminum alloy (*E* = 72.35 GPa, *ν* = 0.337, and *ρ* = 2710 kg/m^3^) and a thickness of 2.4 mm.

## 3. Results and Discussion

### 3.1. Experimental Results

**Figure 4:**
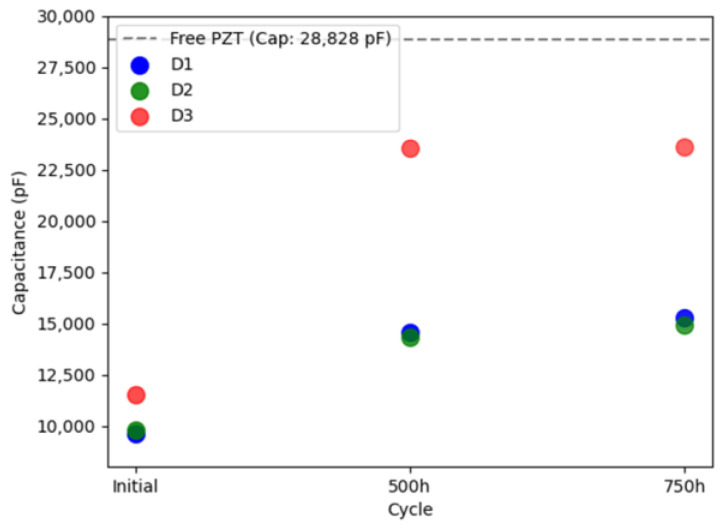
Evolution of the static capacitance of the three PWASs with aging. The dashed line shows the average static capacitance of the free PWAS measured experimentally.

Figure 4 illustrates the evolution of static capacitance for the three examined PWASs. All PWASs consistently exhibited a reduction in static capacitance values compared to their free counterparts. Notably, PWAS D3, initially displaying defective bonding, showed a relatively higher level of capacitance. With subsequent thermal cycling, there was a monotonic increase in capacitance values. The temperature range applied here is gentle enough to not produce meaningful variations in the constitutive piezoelectric properties of a C6 PZT ceramic. Previous studies applying similar thermal cycling [56] reported a reduction in the static capacitance of piezoelectric SHM sensors of ~−9% after 500 cycles. However, it is important to note that the piezoelectric sensor used in those studies differed from the ones analyzed here. In another study [57], minimal yet irreversible changes in the constitutive properties of a soft piezo ceramic (APC 850), somewhat similar to the Fuji C6 used in this work, were reported after exposure to only 50 °C. These changes were attributed to domain wall motion without alterations in the PZT crystal structure. Regardless, the static capacitance values reported herein exhibit an evolution with aging that is likely dominated by modifications in the bonding condition of the PWAS. This assertion is further substantiated through subsequent LDV scan measurements.

**Figure 5:**
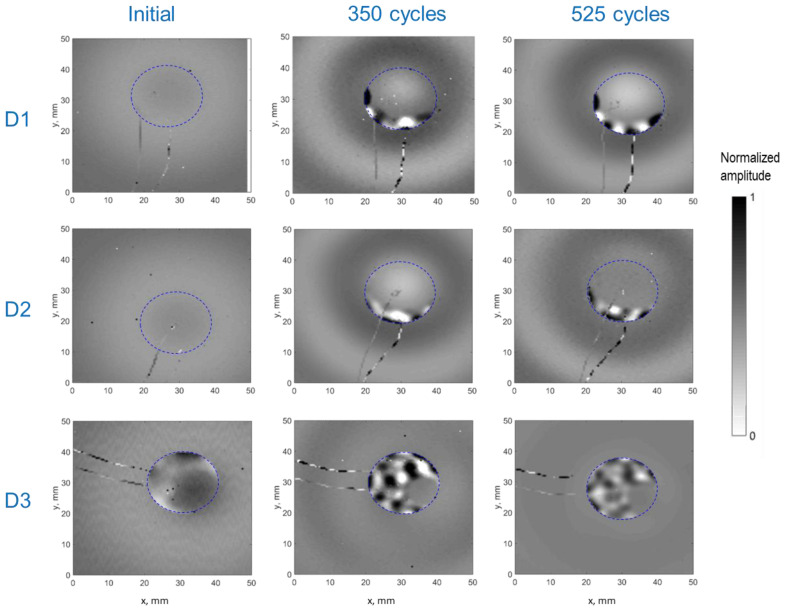
Out-of-plane velocity obtained experimentally for the PWASs D1, D2, and D3 through thermal aging. The dashed line depicts the PWAS perimeter.

Figure 5 compares the out-of-plane velocity scans generated using the PWASs (D1, D2, and D3) at 25 kHz during various aging stages (initial, 350 cycles, and 525 cycles). Note that since absolute values are presented, the minima correspond to the zero-crossing velocity of the waves. Uniformly bonded PWASs (D1 and D2) displayed nearly constant amplitudes across the scanning area, posing a challenge in distinguishing them from the plate. Additionally, a concentric wave field was generated, as was the expectation for a uniformly bonded circular PWAS. Contrariwise, defectively bonded PWASs displayed heightened amplitudes at debonded areas. As aging progressed, all three PWASs started to release at the borders, revealing distinctive resonance-like patterns within the zones, losing adhesion with the aluminum plate. Notably, the PWASs consistently initiated detachment at the position of the cabling electrodes. One potential explanation is stress concentration precisely at the electrode position, possibly influencing subsequent debonding upon thermal cycling. Alternatively, the bonding defects that were initially present could trigger debonding during the process of thermal cycling. These hypotheses require further experimental verification on a larger set of PWASs for validation. Moreover, due to debonding, the generated wave field was no longer concentric to the PWAS’s position, and the amplitude was significantly reduced with increasing debonding area. These results are consistent with previous studies that used LDV scans to examine the health of PWASs bonded to metallic and composite plates [28,45].

**Figure 6:**
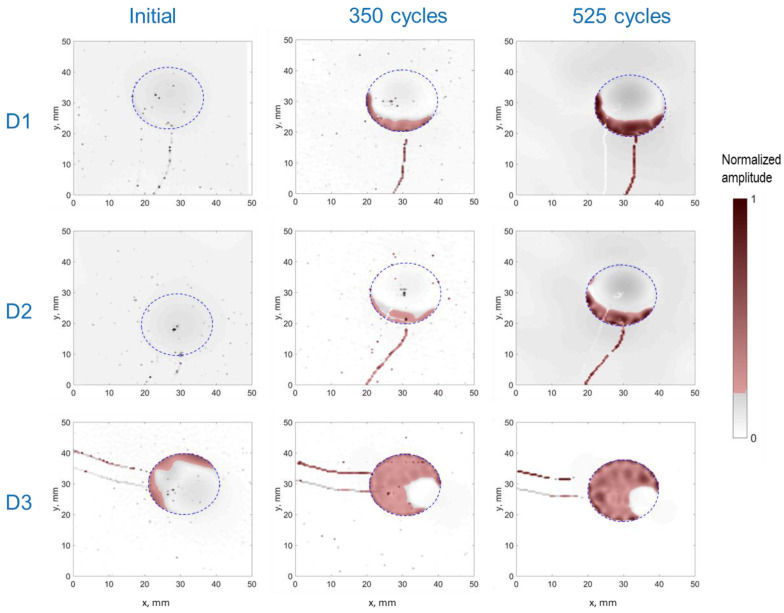
Post-processed RMS amplitude LDV scans obtained for the PWASs D1, D2, and D3 at different ages (the thresholded and binarized images are shown as a red overlay). The dashed line depicts the PWAS perimeter.

**Figure 7:**
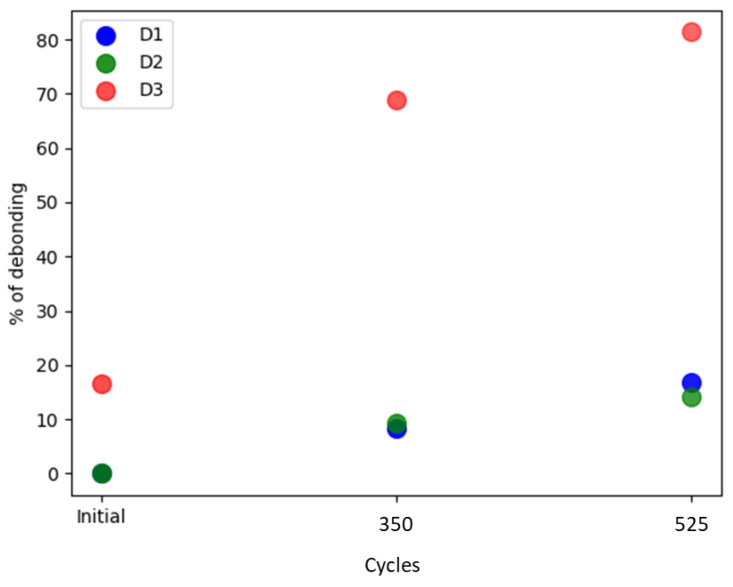
Estimation of the debonded area for all the PWASs according to the filtered RMS amplitudes displayed in Figure 6.

The experimental LDV scans were binarized and thresholded to estimate the debonded area, as detailed in Section 2.2.2. Figure 6 displays the RMS amplitude scans, illustrating the applied filtering process to these scans (note the difference in RMS amplitude representation compared to Figure 5). These results were subsequently used to define the adhesive layer (spring elements) between the PWAS and the aluminum plate in the FEM. Figure 7 provides a quantification of the debonded area for each case based on this filtering approach. Interestingly, the estimated debonded areas exhibit a similar trend to that obtained from the static capacitance measurements (Figure 4), suggesting that the measured static capacitance value scales to the debonding extent. Moreover, according to both the static capacitance measurements and the analysis of the LDV scans, the onset of degradation after 350 cycles was more pronounced for the initially defectively bonded PWAS D3, underscoring the significance of the initial bonding quality on the long-term behavior of the bonded sensors.

### 3.2. FEM Results

**Figure 8:**
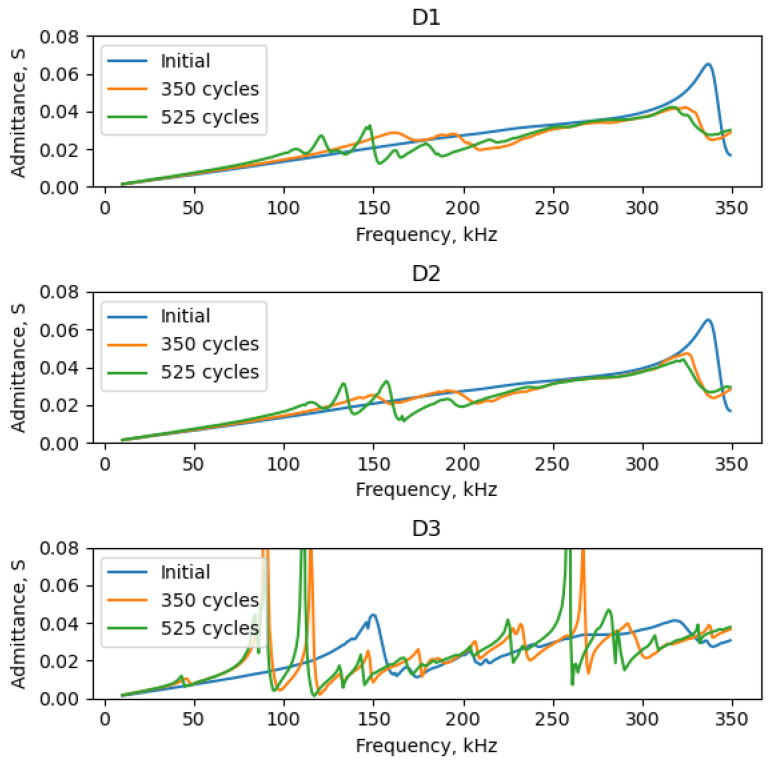
Admittance spectra from FEM simulations: healthy and deteriorated scenarios of the PWASs D1 (**top**), D2 (**middle**), and D3 (**bottom**).

Numerical modeling provided additional insights into the impact of bonding degradation on Lamb waves emitted in a plate. The binarized LDV scans were used to reproduce the different damage scenarios observed during the experimental accelerated aging campaign. In these scenarios, the interfacial elements were selectively positioned solely on the remaining bonded PWAS area. The use of such synthetic scenarios facilitated a more nuanced understanding of the interplay between the bonding conditions and the resultant emitted Lamb waves and PWASs’ admittance spectra. Figure 8 presents three plots, each corresponding to the admittance spectra resulting from the recreated bonding conditions of the PWASs D1, D2, and D3. For perfect bonding conditions, the results indicate that admittance increases proportionally with frequency before the first resonance of the piezoelectric disc (occurring above 300 kHz). In all instances (other than perfect bonding), distinctive resonance peaks are identified, which intensify as the debonding extent increases.

**Figure 9:**
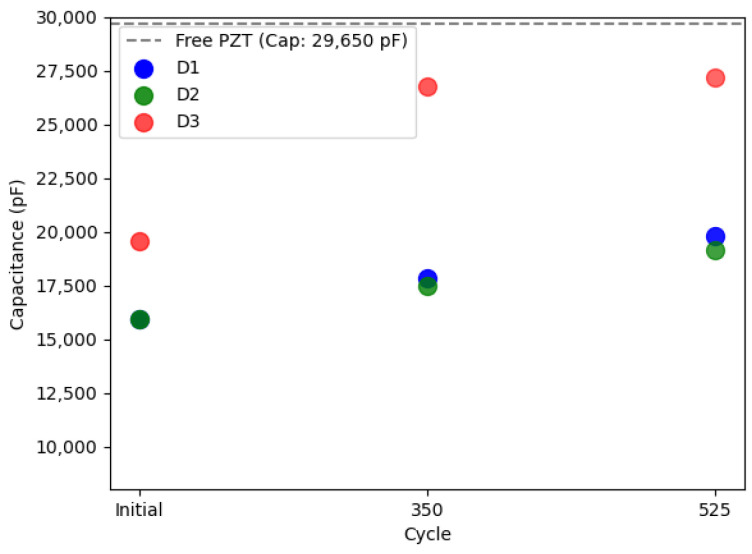
Static capacitance of the three PWASs obtained through the FEM (considering the data sheet properties of the Fuji C6 PZT given in Section 2.3) at the different aging scenarios. The dashed line shows the static capacitance of the free PWAS.

For reference, the static capacitance values of the PWAS obtained from the FEM are shown in Figure 9. Once again, the static capacitance values increase monotonically with the extent of debonding, as was substantiated experimentally. However, the static capacitance values obtained from the FEM were observed to be higher than their experimental counterparts (those shown in Figure 4). In practice, by updating the input parameters of the FEM, similar values can be obtained. Notably, setting the initial adhesive’s stiffness to a higher value (increasing Young’s modulus and decreasing adhesive thickness) and adjusting the constitutive properties of the piezoelectric element and substrate can achieve this. Note that data sheet properties were considered for the PWASs and that the adhesive properties were not experimentally ascertained. Another interesting observation was that the relative changes in static capacitance with thermal cycling obtained experimentally were more prominent than those determined through the FEM. For example, the experimentally measured static capacitance values after 350 cycles for the PWAS D3 exhibited a significant increase, nearly doubling the initial measure. In contrast, the FEM-estimated increase for the same case was only approximately ~35%. Several factors may plausibly account for this observed discrepancy. One is an underestimation of the actual debonded area that was used as an input to configure the adhesive layer in the FEM analysis. Additionally, the evolving physical properties of the adhesive, such as elastic properties and density, are likewise expected to evolve with aging. These changes may progressively result in a less constraining boundary condition on the PWAS and manifest as an increased static capacitance value. Similarly, the constitutive piezoelectric properties of the PWASs may undergo variations with thermal aging. Past studies have documented variations in constitutive piezoelectric properties after exposure to similar temperature conditions. The interplay between these counteracting phenomena during environmental aging cannot be fully apprehended through the sole measure of static capacitance values; so, a more nuanced study is necessary to independently discern the root causes of the observed behavior, some of which are summarized herein. Still, the static capacitance measurement serves as a fundamental diagnostic tool for assessing the bonding quality of PWASs and for detecting deviations from the initial behavior.

**Figure 10:**
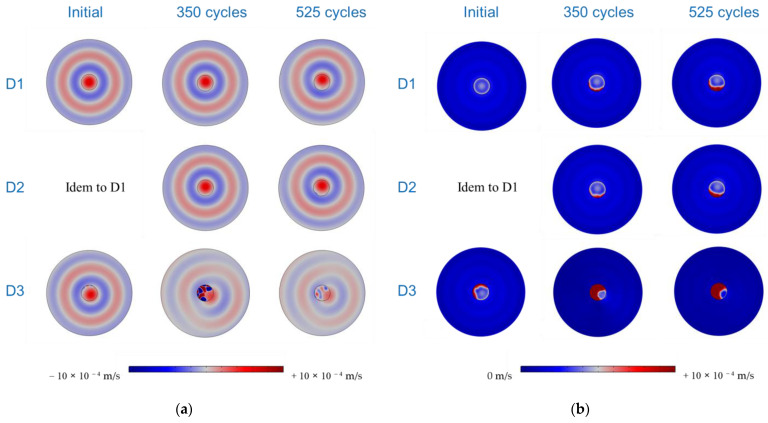
Simulated LDV scans for (**a**) out-of-plane velocity and (**b**) RMS amplitudes, obtained at 25 kHz for all the PWASs and considered damage scenarios.

In Figure 10a,b, the out-of-plane displacement and RMS amplitudes obtained from numerical simulations at 25 kHz (akin to the experimental configuration) are presented. The results exhibit qualitative similarities to the experimental results reported in Section 3.1. The harmonic radiation field for the out-of-plane velocity becomes considerably distorted for the debonded PWAS scenarios, deviating from the expectations for a uniformly bonded PWAS. Moreover, heightened amplitudes and resonance-like patterns also manifest within the debonded PWAS areas, alongside an overall reduction in wave amplitude within the plate.

**Figure 11:**
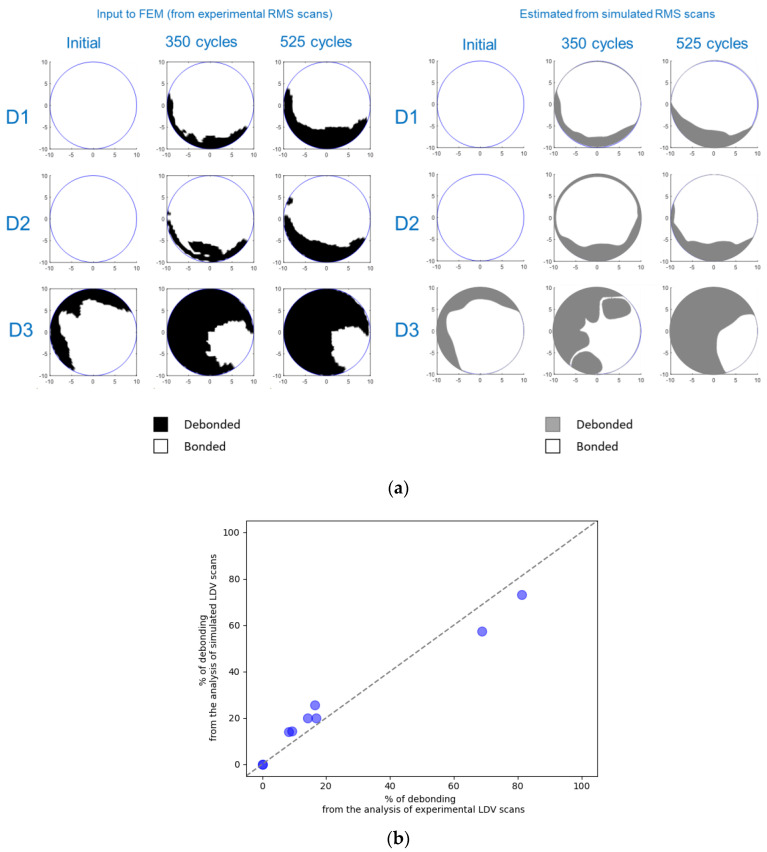
(**a**) Comparison between the input FEM-debonded PWAS areas (retrieved from experimental RMS scans) and their posterior estimates using similar filtering on simulated RMS scans. (**b**) One-to-one comparison of the percentage of debonded area estimates.

An additional analysis was performed to validate the filtering process employed for configuring the adhesive layer in the FEM. Figure 11a,b show the identical filtering process used on the experimental RMS amplitude scans applied to the FEM results (shown in Figure 10b) and compare the one-to-one correspondence between the input debonded area and the output debonded area resulting from the FEM computation. The results indicate that the filtering approach reproduces the debonded area quite well, offering valuable insights into the extent of debonding. Likewise, the shape of the retrieved debonded areas closely matches the input shapes, indicating a fairly good agreement between both of them. Notably, the outcome of this analysis is contingent upon the chosen excitation frequency, which, herein, was arbitrarily set at 25 kHz. Consequently, opting for a different excitation frequency may yield slightly different results, potentially inducing resonance in the PWAS and amplifying the displayed amplitude on its surface. A more comprehensive understanding of the impact of PWAS bonding deterioration can be gained by checking the behavior across a spectrum of frequencies, as was substantiated through the analysis of the admittance spectra. The same allows a more nuanced examination of the effects of deteriorated PWAS bonding on the generated Lamb waves, with a focused exploration of antisymmetric and symmetric modes generated within the plate.

**Figure 12:**
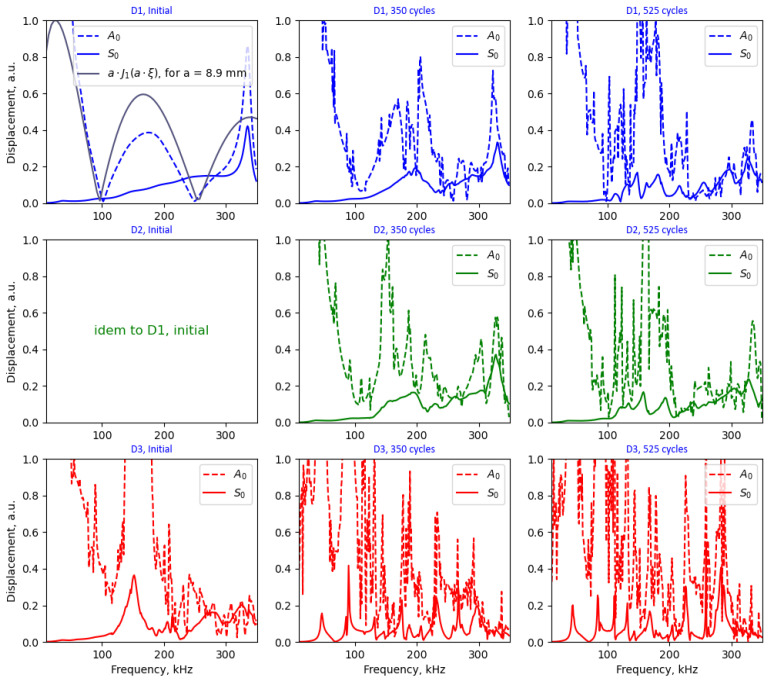
Wave-tuning curves obtained from the FEM at an arbitrary position on the plate for perfect and aged PWAS bonds (retrieved from the post-processed LDV scans shown in Figure 6).

Figure 12 illustrates the wave-tuning curves retrieved at one arbitrary position within the plate for the zeroth-order antisymmetric (A_0_) and symmetric (S_0_) modes. It is important to note that, except for the perfect bond case, the wave-tuning curves are position-dependent on the plate. The FEM results for a perfectly bonded system align with their expected behavior, almost coinciding with the analytical formulae obtained from the mechanistic modeling of a perfectly bonded circular PWAS provided elsewhere [46,58]. According to the formulae provided therein, the amplitude of the A_0_ mode scales to $\left|a\cdot J_{1}\left(\xi a\right)\right|$, where ξ is the wavenumber, and a denotes the PWAS radius. For reference, the analytical result is shown in Figure 12 (only for the perfectly bonded case—PWAS D1). The primary discrepancies between the FEM and the analytical model can be attributed to deviations from the pin force model that forms the basis of the analytical model. As discussed in previous studies [40,58], the validity of the pin force model is perfect when the shear lag parameter used in their formulation approaches infinity. The latter condition is deemed reasonable for very thin and stiff bonding layers. Alternatively, when the shear lag parameter value is small (as is the case in the FEM results presented herein), the force transfer between the PWAS and the plate only takes place over a finite length at the edge of the PWAS, resulting in an effective PWAS dimension smaller than its actual physical one. An effective PWAS radius of 8.9 mm was determined to best fit the wave-tuning curve obtained from the FEM. The results displayed in Figure 12 reveal ‘sweet spots’ for the S_0_ mode near ~100 kHz and ~250 kHz, along with a maximum amplification spot for the A_0_ mode at ~180 kHz. Additionally, a significant observation is the coupling of the first resonance of the bonded PWAS (well above 300 kHz), a phenomenon not predicted by the analytical model.

The results shown in Figure 12 demonstrate that the degradation of the bonding results in a considerable distortion of the wave-tuning curves, aligning with the apparition of PWAS resonances in the admittance spectra (as depicted in Figure 8). The emergence of PWAS resonances generates a complex interplay with the emitted wave and resulting Lamb wave modes in the plate. This phenomenon was already ventured in previous studies [37] and substantiated herein by separately analyzing the A_0_- and S_0_-tuning curves on the plate. Notably, these observations could explain the disparity of pitch–catch results obtained in the literature in what concerns variations in the amplitude and phase of the received waveforms with debonding extent [35,36,37,38]. It is important to note that the tuning curves shown in Figure 12 were obtained for a specific position on the plate, and they differed for other positions. This suggests that the emitted waves undergo a significant level of variation with debonding and that the results are dependent on the relative position of the debonding along the propagation pitch–catch path.

## 4. Conclusions

Experimental and numerical investigations were conducted on PWASs bonded to an aluminum plate, aiming to understand the impact of bonding degradation on Lamb wave generation. In the experiments, three surface-bonded PWASs were analyzed, with one intentionally bonded with a lower amount of adhesives to create a defective bond. All three PWASs underwent thermal cyclic aging between −55 °C and 85 °C, and LDV and static capacitance measurements were used to monitor degradation. The PWAS with the initially defective bond demonstrated the least favorable performance throughout the aging cycles, underscoring the significance of the initial bond condition on their long-term behavior. In all instances, as the debonding extent increased, the electromechanical behavior was modified, as substantiated by the static capacitance measurements and LDV scans. While this study specifically addressed the effects of PWAS debonding, it is anticipated that environmental testing may induce variations in the constitutive piezoelectric properties of the PWAS elements, as well as alterations in the physical properties of the adhesive layer. These changes can collectively impact the electromechanical behavior of the bonded PWAS. The interplay between these counteracting phenomena may result in complexities that are challenging to grasp solely through electrical impedance measurements.

The purpose of the FEM was to simulate the effects of a debonded PWAS on its admittance spectrum and investigate the radiation field produced by the PWAS actuating as an emitter when accounting for a realistic representation of the actual debonded area at different aging states. The analysis of a perfectly bonded PWAS aligned with analytical modeling has been provided elsewhere [58]. A key consequence supported by both numerical simulations and experimental observations is the reduction in wave amplitude and notable distortion of the generated wave field. Another discernible effect is the deviation of the wave field on the plate from the concentric radiation pattern anticipated for a perfectly bonded circular PWAS. The emergence of PWAS resonances upon debonding resulted in a complex variation in Lamb wave modes amplitudes that challenges the validity of analytical wave-tuning formulae. In this context, it becomes apparent that assessing PWAS bond degradation through pitch–catch measurements may lead to misleading conclusions. Such evaluations strongly depend on the wave path and the relative position of the debonding within the emitting and receiving PWAS. Regardless, the ultimate implication of these observations is a loss of function, affecting the probability of detection and localization and potentially leading to false positives. The latter was recently substantiated using a FEM of a sparse array of perfectly bonded PWASs that was then compared to scenarios involving deteriorated bonding. The results revealed a diminished ability to localize damage, which, in turn, depends on the applied localization algorithm [59].

As a final remark, the current SAE ARP6461 underscores the imperative that, for proper functioning, SHM sensors necessitate a high-integrity bond with the monitored structure, and such integrity must be upheld throughout the structure’s lifetime. However, solely relying on mechanical means to establish criteria for sensor survivability may not be sufficient to prevent SHM dysfunction. Currently, there is no consensus on features or standard experimental procedures to establish deemed-to-satisfy criteria for assessing the remaining performance of SHM systems. This challenge is further compounded by the complexity of accurately reproducing the intricate combination of stresses present in real aircraft environments through accelerated testing. Additionally, quantitatively assessing the performance of structural health monitoring (SHM) systems requires the use of the appropriate metrics. The latter was also recently highlighted by Falcetelli et al. [60]. Our research group is currently conducting ongoing studies to quantify the performance of structural health monitoring (SHM) systems. The anticipated outcome of this research effort is expected to boost confidence in the use of bonded PWASs, as well as other sensors for aerospace SHM applications, while offering valuable insights into the remaining lifetime of SHM systems. Despite our focus on aircraft applications herein, these considerations are also relevant to SHM applications beyond the aerospace industry.

## Data Availability

The data are available upon a reasonable request.
